# Dynamic expression of synemin isoforms in mouse embryonic stem cells and neural derivatives

**DOI:** 10.1186/1471-2121-12-51

**Published:** 2011-11-23

**Authors:** Sheila C de Souza Martins, Onnik Agbulut, Nicolas Diguet, Jean-Christophe Larcher, Bruna S Paulsen, Stevens K Rehen, Vivaldo Moura-Neto, Denise Paulin, Zhenlin Li, Zhigang Xue

**Affiliations:** 1 Department of Aging, Stress and Inflammation, UPMC Sorbonne University, Paris, France; 2 Instituto de Ciências Biomédicas, Universidade Federal do Rio de Janeiro, Rio de Janeiro, Brasil; 3 Department of Functional and Adaptive Biology, Paris Diderot University, Paris, France; 4 Department of Developmental Biology, UPMC Sorbonne University, UMR 7622 CNRS, Paris, France

## Abstract

**Background:**

Intermediate filaments (IFs) are major components of the mammalian cytoskeleton and expressed in cell-type-specific patterns. Morphological changes during cell differentiation are linked to IF network remodeling. However, little is known concerning the presence and the role of IFs in embryonic stem (ES) cells and during their differentiation.

**Results:**

We have examined the expression profile of synemin isoforms in mouse pluripotent ES cells and during their neural differentiation induced by retinoic acid. Using RT-PCR, Western blotting and immunostaining, we show that synemin M is present at both mRNA and protein levels in undifferentiated ES cells as early as pluripotency factor Oct-3/4 and IF keratin 8. Synemin H was produced only in neural precursors when neural differentiation started, concurrently with synemin M, nestin and glial fibrillary acidic protein. However, both synemin H and M were restricted to the progenitor line during the neural differentiation program. Our *in vivo *analysis also confirmed the expression of synemins H/M in multipotent neural stem cells in the subventricular zone of the adult brain, a neurogenic germinal niche of the mice. Knocking down synemin in ES cells by shRNA lentiviral particles transduction has no influence on expression of Oct4, Nanog and SOX2, but decreased keratin 8 expression.

**Conclusions:**

Our study shows a developmental stage specific regulation of synemin isoforms in ES cells and its neural derivatives. These findings represent the first evidence that synemins could potentially be useful markers for distinguishing multipotent ES cells from undifferentiated neural stem cells and more committed progenitor cells.

## Background

Synemin belongs to the intermediate filament (IF) protein family [1,2]. In mammals, the synemin gene is one of the rare IF genes encoding three isoforms (180, 150 and 41 kDa) achieved by alternative mRNA splicing, exon skipping and a shift in the open reading frame [3,4]. The two larger isoforms of synemin (H and M) harbor extended C-terminal tails that project from the surface of the filament and provide connecting arms that associate with neighboring proteins [5-7]. In contrast, the small isoform (L) lacks this tail domain [3,8].

Synemin forms obligatory heteropolymers in order to incorporate into filamentous networks and is associated with desmin and vimentin in muscle and endothelial cells [1-3,9]. It has been suggested that synemin could function as a linker between different cytoskeletal components based on the fact that it interacts with several proteins involved in the organization of the costameres, neuromuscular and myotendinous junctions within striated muscle cells. These proteins include α-actinin, vinculin, dystrophin, α-dystrobrevin, utrophin, zyxin and talin [1,3-7,10-13]. In addition, it has been reported that synemin is an A-kinase anchoring protein (AKAP), containing a binding region for protein kinase A (PKA) in its C-terminal domain which provides temporal and spatial targeting of PKA in cardiomyocytes [14]. In the nervous system, synemin was found to associate with ruffled membranes of reactive astrocytes and to also co-localize with α-actinin, suggesting a role in cell motility [15]. We have shown that synemins H and M were present with vimentin, nestin and glial fibrillary acidic protein (GFAP) in glial progenitors, whereas the small isoform appeared in neurons linked to the NF proteins associated with the membrane compartment [9,16].

The different expression of isoforms H, M and L of synemin in the nervous system raises several questions about the regulation of synemin gene expression during the determination of glial and neuronal cell lineages in the central and the peripheral nervous system (CNS and PNS). First, an unexpected finding is the selective synthesis of two high molecular weight synemin isoforms (H and M) in CNS astrocytes, while the smallest synemin isoform (L) is present only in neurons [16]. This selectivity suggests that the commitment of CNS precursor cells to form glia or neuron involves the direct regulation of the single synemin gene. Our analysis of mouse development from embryonic day 5 to 15 (E5 to E15) has demonstrated that synemin M mRNA is produced at E5 as early as nestin and vimentin mRNA, prior to the appearance of the H isoform. *In toto *hybridization at E7.5 showed synemin M labeling in the embryonic mesoderm and the neuroectoderm [9]. We asked if synemin is also expressed in mouse embryonic stem (ES) cells, which can differentiate into a variety of somatic cell types including lineages from three embryonic germ layers? The presence and function of IF proteins in ES cells are not yet fully understood, only a few members of this family have been studied [17-20]. The mRNAs coding for keratins 7, 8, 18 and 19 are present in the 2-cell stage embryo, but only K7 and K8 (type II) are translated into protein in 4- to 8-cell stage embryos at a low level, which is deposited in aggregates [18,19,21-23]. The K18 and K19 proteins were identified from E3.5 mouse embryo [19,24]. After differentiation of ES cells into neuronal progenitors, K8 and K18 protein expression decreased [24]. The nuclear IF protein lamin B1 and B2 were identified as markers of ES cells; however the lamins A/C were activated during ES cell differentiation [25].

In this report, we examined the expression profile of synemin isoforms in mouse pluripotent ES cells and during their early neural differentiation induced by retinoic acid to answer the following questions: 1) Are synemin isoforms expressed in pluripotent ES cells? 2) How are synemin isoforms expressed during neural differentiation of ES cells? We detected the expression of synemin M at both mRNA and protein levels in pluripotent ES cells, as early as Oct-3/4 and keratin 8. Synemin H was produced only in neural precursors when the neural differentiation started, along with synemin M, nestin and GFAP.

## Results

### Synemin M expression in mouse pluripotent ES cells

**Figure 1:**
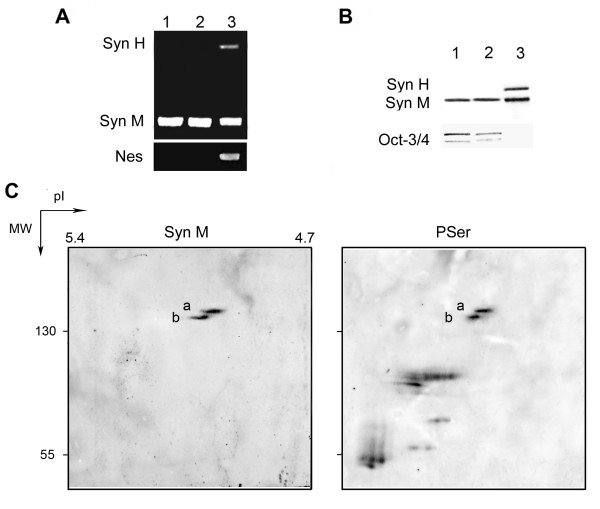
**Characterization of synemin M in mouse ES cells**. A, Analysis by RT-PCR of mRNA encoding synemins H and M isoforms and nestin in USP-1 (lane 1), CGR8 (lane 2) and mouse embryo at E9.5 (lane 3). Synemin M mRNA was found in pluripotent ES cells, while synemin H and nestin mRNAs were absent (lanes 1 and 2). Both synemin isoforms were detected in the 9.5-day-old embryo (lane 3). B, Characterization of synemins H, M and Oct-3/4 proteins by Western blot. USP-1 (lane 1), CGR8 (lane 2) ES cells and the adult mouse bladder (lane 3) were used for this study. Only the synemin M and Oct-3/4 were found in pluripotent ES cells (lanes 1 and 2). The synemin H was absent from undifferentiated ES cells (lanes 1 and 2). The adult mouse bladder was used for a control (lane 3). C, Analysis of synemin in mouse ES cells by 2D-PAGE and immunoblotting. Total protein extract of ES cells was separated by using pH 4-6 gradients IPG strips and 8% SDS/PAGE gels. Two spot trains of synemin M with isoelectric points (pI) 5.1-5.2 and apparent 148-150 kDa molecular weights (MW) were characterized. Analysis of the phosphorylation of synemin M revealed that two spot trains of synemin M contained phosphoserines (PSer) with isoelectric variants. Syn: synemin.

To investigate which synemin isoforms are expressed in ES cells, two mouse ES cell lines were used. We first analyzed the pluripotent state of the cells by PCR-Array. We identified the expression of transcription factors maintaining "stem-ness" as Nanog, Nr6a1, Utf1, Pou5f1 (Oct-3/4), and signaling molecules required for pluripotency and self-renewal genes such as Ifitm1 and Pten. No expression of embryonic stem cell differentiation genes was detected in the ES cells (Data not shown). Expression of synemin mRNA in ES cells was analyzed by RT-PCR. As shown in Figure 1A, we detected the synemin M mRNA in ES cells. In contrast, neither synemin H nor nestin mRNA was found. The E9.5 mouse embryos containing synemin M and H were used as a positive control (Figure 1A).

The presence of synemin M protein, but not synemin H, was confirmed by Western blot analysis in ES cells (Figure 1B). In the positive control, both synemin M and H isoforms were identified in the adult mouse bladder. The presence of Oct-3/4, a marker of the pluripotency of ES cells, was also confirmed by Western blotting (Figure 1B). Two-dimensional polyacrylamide gel electrophoresis (2-D PAGE) followed by Western blotting was used to further analyze the synemin profile from ES cells extracts. This experiment showed two spot trains (a and b) of synemin M with an apparent molecular weight of 148-150 kDa and isoelectric point (pI) 5.1-5.2 in each ES cell line (Figure 1C), corresponding to its theoretical pI (5.14), in agreement with its high content in glutamic acid and glutamine (18%, 228 residues/molecule), aspartic acid (6%, 70 residues/molecule), and rich in serine (9%, 111 residues/molecule) and poor in cysteine (0.1%, 2 residues/molecule). We could observe that the quantity of spot train-a of synemin M was more abundant than spot train-b. In order to investigate the possible post-translational modifications of synemin, we analyzed the phosphorylation of synemin M in ES cells with anti-phosphoserine antibody. This result revealed that synemin M was phosphorylated in serine with isoelectric variants (Figure 1C).

**Figure 2:**
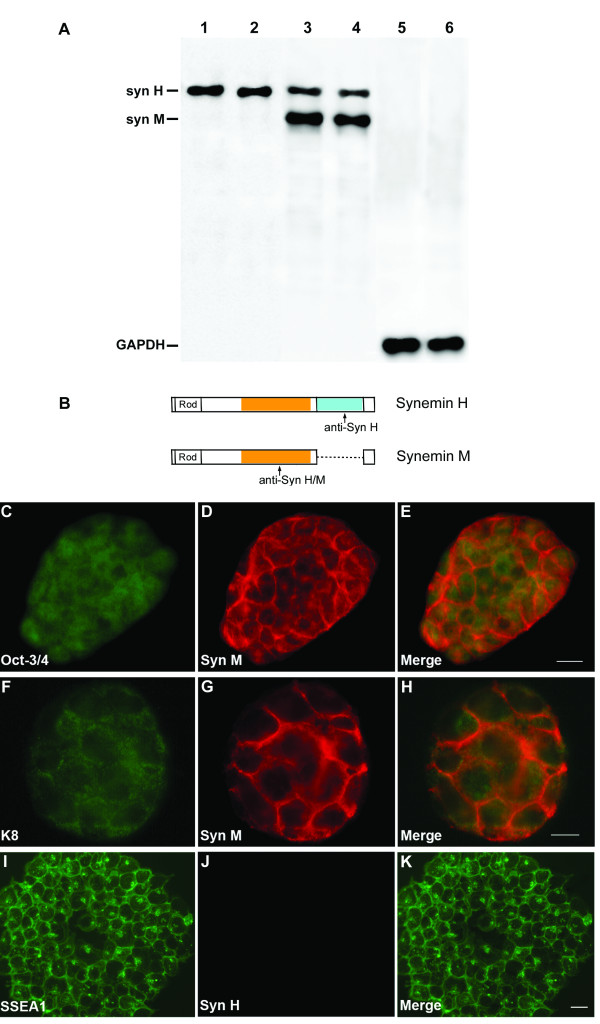
**Immunofluorescence detection of synemin in mouse ES cells**. A, Western blotting showing the specificity of synemin antibodies with proteins extracted from mouse bladder. The antibody anti-synemin H detected one band of 180 kDa, corresponding to synemin H isoform (lanes 1-2), and the anti-synemin H/M detected two bands 150 kDa and 180 kDa that correspond to H and M isoforms (lanes 3-4). GAPDH was used for the loading control (lanes 5-6). B, Diagram illustrating the regions recognized by antibodies anti-synemin H and anti-synemins H/M. C-K, Immunochemistry analysis demonstrating ES cells colonies labeled for Oct-3/4 (C), keratin 8 (F), SSEA-1 (I), synemin M (D, G) and synemin H (J). Synemin M was present with Oct-3/4 (C-E) and keratin 8 (F-H) in pluripotent ES cells. No immunolabelling for synemin H was observed in undifferentiated ES cells expressing SSEA-1 (I-K). Syn: synemin; K8: keratin 8. Bar = 20 μm.

To determine the synemin localization in ES cells, we performed double immunofluorescence staining utilizing synemin, Oct-3/4, cytokeratin 8 (K8), and SSEA-1 antibodies. In order to study specifically the expression of the synemin H isoform, we produced a polyclonal antibody with a polypeptide corresponding to the exon 4a of human synemin, a specific fragment for synemin H [4]. Western blotting analysis with proteins extracted from the mouse bladder showed that this antibody recognized specifically one band of 180 kDa, corresponding to the synemin H isoform (Figure 2A). The immunocytochemistry study clearly showed the presence of the synemin M protein in pluripotent ES cells expressing Oct-3/4 (Figures 2C-E) and K8 (Figures 2F-H). However, synemin H was never detected in undifferentiated ES cells (Figure 2J) expressing the pluripotent ES marker such as SSEA-1 (Figures 2I-K). It is remarkable that neither synemin nor keratin was organized in filamentous IF networks in pluripotent ES cells due to the absence of their partners.

### Change of synemin expression profile during neural differentiation of ES cells

**Figure 3:**
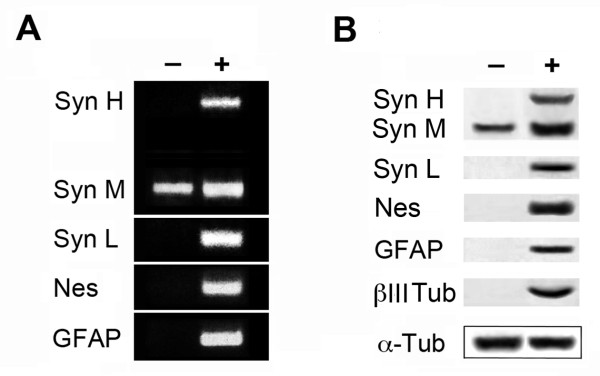
**Analysis of synemin isoforms after neural differentiation of mouse ES cells**. A, RT-PCR analysis of mRNA encoding synemins H, M and L isoforms, nestin, and GFAP in retinoic acid (RA) treated (**+**) and control (**-**) EBs. Synemin M mRNA was found in both control and differentiated ES cells, while synemin H, synemin L, nestin and GFAP mRNA were found only in differentiated ES cells (**+**) and absent in control ES cells (**-**). B, Western blotting analysis of synemins (H, M and L), nestin, GFAP and β-III tubulin proteins extracted from RA treated (**+**) and control (-) EBs. Synemins H and L, nestin, GFAP, and β-III tubulin proteins were detected in retinoic acid treated (**+**) EBs and absent in control (**-**) EBs. An increase of synemin M protein was observed in EBs after retinoic acid treated (**+**). α-tubulin was used for the loading control. Syn: synemin; Nes: nestin; βIII Tub: β-III tubulin; α-Tub: α-tubulin.

Since synemin isoform expression during the mouse embryo development is complex [9], we decided to study the possible change of synemin isoform expression profile during ES cell neural differentiation. To achieve this, cultured embryoid bodies (EBs) were treated with retinoic acid (RA), an inducer of neural differentiation [26-28]. The characteristics of differentiated ES cells were then analyzed by RT-PCR, Western blotting and immunochemistry. The expression of synemin H mRNA as well as synemin L mRNA was detected in differentiated ES cells along with synemin M, nestin, a specific marker of neural precursor cells [29,30], and GFAP, a specific marker of the glial cell lineage [31-34] (Figure 3A). However, the accumulation of synemin H mRNA was less abundant than synemin M (the ratio of two isoforms is about 5). The appearance of these neural markers (nestin, GFAP, and β-III tubulin) in RA treated EBs was also confirmed by Western blotting (Figure 3B). As shown in Figure 3B, the induction of synemin H and synemin L was observed in differentiated ES cells after RA treatment as well as an increase of synemin M.

**Figure 4:**
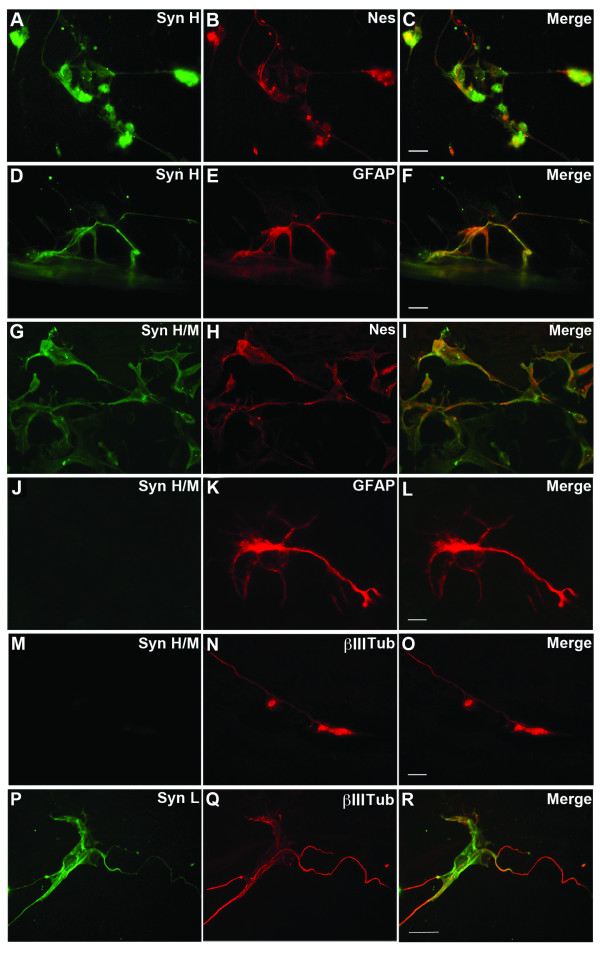
**Synemins H and M distribution during neural differentiation of mouse ES cells**. Retinoic acid-treated EBs were immunolabelled for synemin H (A, D), synemin H/M (G, J, M), synemin L (P), nestin (B, H), GFAP (E, K) and β-III tubulin (N, Q). The heterogeneity of differentiated ES cells was observed. Synemin H and M were co-expressed with nestin in a subpopulation of neural precursors (A-C, G-I), also with GFAP in a subpopulation of glia-astrocyte precursors harboring low level of GFAP (D-F). In contrast, synemin expression became extinct when astrocytes expressing high level of GFAP (J-L). During neuronal differentiation, no synemin H or M was detected in the neuronal cell expressing β-III tubulin (M-O). However, synemin L was co-expressed with β-III tubulin in neurons (P-R). Syn: synemin; Nes: nestin; βIII Tub: β-III tubulin. Bar = 20 μm.

When RA was added to EBs cells, the distribution of synemin H and M was limited to the inner cells of the EB and to a subpopulation of cells that migrated from it, presumably radial glia cells. Differentiated ES cells presented an evident heterogeneity. We observed that almost all of the neural precursors (90%) expressing synemin H and synemin M are nestin positive (Figures 4A-C, G-I). The expression of synemin H in differentiated ES cells was always associated with synemin M. During glial cell differentiation, synemin H and M were expressed in the immature astrocytes cells harboring low level of GFAP (Figures 4D-F and data not shown). In contrast, synemin H/M expression became extinct when astrocytes express high level of GFAP (Figures 4J-L).

The neuronal differentiation in EBs was also observed after RA treatment. These neuronal cells positive to β-III tubulin did not contain the synemin H/M (Figures 4M-O). However, the antibody directed against synemin L reacted with the neurons expressing β-III tubulin (Figures 4P-R).

**Figure 5:**
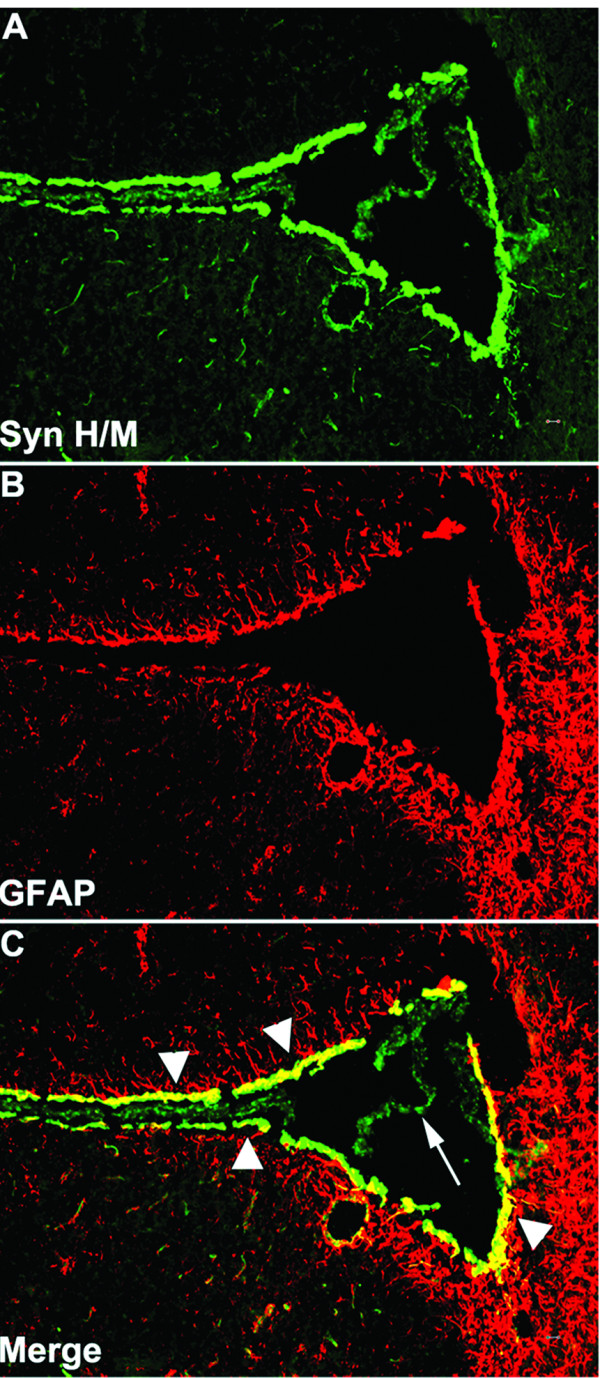
**Synemins H and M distribution in adult mouse subventricular zone *in vivo***. Sections from adult mouse brain were stained with antibodies against synemins H and M in green (A) or GFAP in red (B). Synemins H/M were detected in the limited neural stem cells of the subventricular zone (SVZ, arrowhead) and the choroid plexus (arrow) of the adult mouse brain. GFAP was present in neural stem cells of the SVZ and also in mature cortical astrocytes. Overlay in C shows the co-localization of synemin with GFAP in the neural stem cells.

Our *in vivo *analysis in the adult mouse brain also confirmed the expression of synemins H and M in multipotent neural stem cells. These synemin isoforms were detected only in multipotent neural stem cells, particularly in the subventricular zone of the lateral ventricle (Figure 5A), a neurogenic germinal site in adult mice [33,35]. However, GFAP was present in these neural stem cells as well as in mature cortical astrocytes (Figure 5B).

### Synemin knockdown in mouse ES Cells by shRNA lentiviral particles

**Figure 6:**
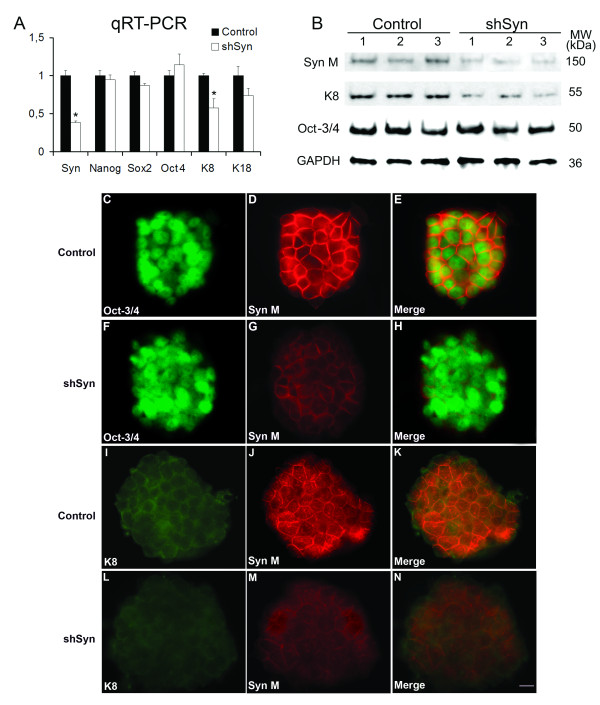
**Synemin knockdown in mouse ES cells by shRNA lentiviral particles**. A, Analysis of mRNA expression by qRT-PCR. Messenger RNA was extracted from ES cells infected by control or synemin shRNA lentivirus (shSyn). The PCR analysis was performed with SYBR green PCR technology. HPRT was used as a control gene for normalization. B, Western blotting analysis of synemins M (Syn M), Keratin 8 (K8) and Oct-3/4 proteins extracted from ES cells infected by control or synemin shRNA lentivirus (shSyn). GAPDH was used for the loading control. C-N, Immunochemistry analysis demonstrating ES cells colonies labeled for Oct-3/4 (C, F), keratin 8 (I, L) and synemin M (D, G, J, M) in mouse ES cells infected by control (C-E, I-K) or synemin shRNA lentivirus (shSyn, F-H, L-N). Synemin knockdown in mouse ES cells by shRNA lentivirus has no influence on expression of Oct4, but decrease keratin 8 expression. Bar = 20 μm.

**Figure 7:**
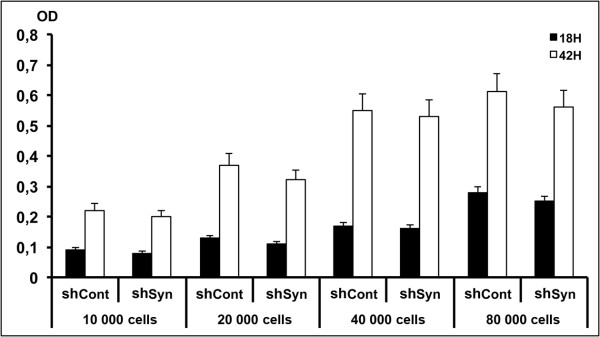
**MTT assay was used to determine the effect of synemin knockdown on mouse ES cell proliferation activity at 18 and 42 hours culture**. No significant modification of cell proliferation in the shSyn group when compared to the control group. OD: optical density.

To test whether synemin expression is functionally required in ES cells, we carried out a synemin knockdown experiment in mouse ES cells by shRNA lentiviral particles transduction. The transduced mouse ES cell clumps were selected via Puromycin dihydrochloride selection, and then analyzed by qRT-PCR, Western blot and immunocytochemistry. This study showed that knocking down synemin (by 61.5%) decreased the expression of keratin 8 by 42.6% at mRNA level (Figure 6A). A similar decrease was also detected by Western blotting (Figure 6B) and immunocytochemistry analyses (Figures 6I-N). Blast program (NCBI) analysis indicates that three synemin siRNA sequences used in this experiment do not share any homology with other genes, so K8 decrease in this study is unlikely due to the direct inhibition of synemin siRNA. The relationship between synemin and K8 in ES cells remains to be studied. Synemin knockdown in mouse ES cells did not influence the expression of Oct4, Nanog and SOX2 (Figures 6A-B). This result was also confirmed by immunocytochemistry study (Figures 6C-H). In order to determine whether synemin knockdown can affect the growth ability of mouse ES cells, MTT assay was used to test the changes of cell growth ability. As seen in Figure 7, there is no significant modification of cell proliferation in the shSyn group when compared to the control group.

## Discussion

The exact mechanisms underlying the developmental changes in stem cell and precursor population are not understood. The adult vertebrate CNS consists of four major differentiated cell types: neurons, astrocytes, oligodendrocytes and ependymal cells. How these differentiated cell types are generated during early embryogenesis remains mostly unknown due to the complexity of cell structure in this system, the lack of specific cell surface markers to identify distinct cell types and the presence of numerous amplifying cell population in transit that rapidly generate early progenitors. We demonstrated here that synemin expression is characteristic of mouse ES cell lines in the undifferentiated state and the neural stem cells at both mRNA and protein levels. Our experiment showed that mouse pluripotent ES cells expressed synemin M, prior to nestin, which is considered as a marker of neural stem cells [20,29,30]. Moreover, this expression was associated with a common transcriptional signature that is shared by stem cells. This signature includes the expression of stem cell markers, such as homeobox protein Nanog, a transcription factor that functions in concert with other factors such as Oct4 and SOX2 to establish the self-renewal of undifferentiated ES cells [36-38], Oct4, a transcription factor restricted to totipotent and pluripotent cells [39-41], and SSEA-1, an ES cell surface biomarker [42]. It has been reported that there is a strong correlation between Oct4 gene expression and synemin regulation. Knocking down Oct4 by RNAi resulted in a dramatic decrease of synemin M [40]. Synemin knockdown in mouse ES cells by shRNA lentiviral particles did not modify the expression of Oct4, Nanog and Sox2 (Figures 6A-B) showing that synemin can not influence these transcriptional factors. As Oct4 is required to maintain the pluripotentiality and self-renewal of ES cells and since synemin M expression is under its regulation, this suggests that synemin M could be implicated in ES cell maintenance. Synemin knockdown did not influence the ES cell growth. The exact nature of how synemin is implicated in the maintenance of mouse ES cells remains to be studied.

Cytoskeletal proteins play important regulatory roles in a variety of cellular processes including proliferation, migration and differentiation. Among the three types of cytoskeletal elements, two are clearly present in mouse oocytes: microtubules and microfilaments, the presence of IFs being more controversial. The organization and role of the cytoskeletal networks (mainly microtubules and microfilaments) during oogenesis, fertilization and pre-implantation development of the mouse are described given the importance of cell-cell interactions and of the subcellular organization in events leading to the formation of the first two lineages of the mouse embryo [43]. In recent years, IFs have attracted much interest, largely because their constitutive polypeptide units are specifically expressed in various cell types and thus represent excellent differentiation markers [17,44]. However, the presence of IFs in ES cells is not clear, and there is no evidence supporting a role for IF proteins in ES cell. Cytokeratins seem to be the first IFs expressed in embryogenesis [18,19]. Our findings represent the first evidence that synemin M could be a novel characteristic of stem cells. Our functional study demonstrated that synemin knockdown in ES cells by shRNA lentiviral particle transduction also decreased keratin 8 expression. However, their correlation in ES cells is still not clear. In addition, our protein analysis showed that pluripotent ES cells contain two spot groups with isoelectric variants of synemin M (Figure 1C). These isoelectric variations of synemin M may reflect differences on its phosphorylation level. In fact, we have observed that two spot trains of synemin M were phosphorylated (Figure 1C). It is known that IFs are among the most abundant cellular phosphoproteins, their physiologic phosphorylation is typically heterogeneous and implicated in the structural organization of IFs [2,45]. Early studies indicated that synemin provides temporal and spatial targeting of protein kinase A (PKA) in adult and neonatal cardiac myocytes [14]. PKA targeted to IFs could phosphorylate its substrates and it was previously reported that the synemin avian ortholog is phosphorylated by PKA [46]. Moreover, synemin itself may be regulated through phosphorylation by the catalytic subunit of PKA [14]. It is also noteworthy that the cAMP/PKA pathway plays an important role in murine ES cell self-renewal pathways. Directly inhibiting PKA activity in the presence of LIF causes a decrease in Oct4 expression; however, in the absence of LIF, there is increased Oct4 expression [47]. The further investigation may be particularly attractive to identify the mechanism by which the post-translational modification of synemin protein is taken place during stem cell differentiate.

During neural differentiation of ES cells evoked by RA-treatment, the expression of synemin H was induced in neural stem cells along with synemin M, nestin and GFAP expression, which characterizes synemin H as a potential marker of the neural stem cell differentiation. Our *in vivo *analysis also confirmed the expression of synemins H and M in multipotent neural stem cells in the subventricular zone of the adult mouse brain, a niche of the neural stem cells [33,35]. Furthermore, in agreement with our previous data, synemin L is expressed only in mature neurons [9,16]. This selectivity suggests that the commitment of neural precursors to form astroglia or neuron as early as stem cell differentiation involves the direct regulation of the single synemin gene. We therefore believe that the production of synemin proteins is closely associated with the early specialization of ES cells.

## Conclusions

The work presented here demonstrates a complex regulation of synemin gene expression and a developmental stage specific production of each isoform in ES cells and during their neural differentiation. The expression of synemins is characteristic of mouse ES cell lines in the undifferentiated state and the neural stem cells. Synemin M was present in pluripotent ES cells, together with Oct-3/4 and SSEA-1, prior to nestin, a known marker of neural stem cells. Synemin H is up regulated during retinoic acid induced neural differentiation and allows a distinction between an ES cell state and a neural committed stage. During neural differentiation there was also a distinct expression of synemin H, M and L. Synemin H and M are specific for the glial lineage during neural differentiation, whereas synemin L characterized only neuronal cells. The synemin knockdown in ES cells has no influence on expression of Oct4, Nanog and SOX2, but decreased keratin 8 expression. Taken together, the findings herein represent the first evidence that synemins might offer potentially novel and useful markers for distinguishing multipotent ES cells from undifferentiated neural stem cells and more committed progenitor cells.

## Methods

### Cell culture

Two mouse ES cell lines were used during our work: USP1 [48] and CGR8 [22]. USP1 ES cells were maintained in an undifferentiated state on mouse embryonic fibroblast (MEF) feeder layer, mitotic inactivated with mitomycin C (Sigma-Aldrich), in DMEM/F12 with 15% (v/v) knockout serum replacement, 2 mM L-glutamine, 1% (v/v) nonessential amino acids, 0.1 mM β-mercaptoethanol (Invitrogen), 40 μg/mL gentamicin sulfate (Scheing-Plough) and 1000 U/mL of leukemia inhibitory factor (LIF, Invitrogen). CGR8 murine ES cells were cultured on 0,2% (w/v) gelatin (Sigma) coated tissue culture plates in Glasgow's modified Eagle's medium supplemented with 10% (v/v) fetal calf serum, 2 mM L-glutamine, 1% (v/v) nonessential amino acids, 0.1 mM β-mercaptoethanol, 1 mM sodium pyruvate, 50 U/mL penicillin, 50 mg/mL streptomycin (Invitrogen). 1000 U/mL of LIF were added prior to use. All cultures were incubated at 37°C in a humidified 5% CO_2_/95% air.

### Formation and treatment of embryoid bodies (EBs)

A standard protocol for EB formation was used. Briefly, USP1 ES cells and MEF cell monolayers were dissociated with TrypLE™ Express (Invitrogen) and were plated on gelatin-treated dishes for 30 min to remove the feeder cells. This procedure was repeated twice. CGR8 ES cells were used directly. For EBs formation, ES cells (4 × 10^5^/mL) were passed to a 6-well plate covered with 2% (w/v) gelatin (Sigma) and cultured for 48 h (d0-d2) in ES medium as described above. Colonies were then treated with TrypLE™ Express for 5 minutes at 37°C, transferred to non-adherent plate dishes (2.5 × 10^5^/mL) with EB medium (DMEM/F12 medium with 15% (v/v) fetal bovine serum (Invitrogen), 2 mM glutamine, 1% (v/v) nonessential amino acids, 0.1 mM β-mercaptoethanol, 50 U/mL penicillin, 50 mg/mL streptomycin without LIF). ES cells were plated as hanging drops on a lid of 10-cm non-adherent Petri dish. The lid was inverted over the bottom of the dish filled with culture medium. Spherical cell aggregates (EBs) were formed during 4 days (d3-d6). The resulting EBs were transferred into Petri dishes (10-15 EBs per 6-cm dish) in 4 mL of the EB medium, and treated with all-trans-retinoic acid (RA, Sigma-Aldrich) during additional 4 days (from d7 to d10) to induce neural differentiation. At d7, EBs were divided in 2 groups: a) 0.02% (v/v) vehicle (DMSO) and b) 2 μM RA. The medium was changed every two days.

For studying the neuronal differentiation, EBs were plated onto 1 μg/mL laminin (Invitrogen) and 1 μg/mL fibronectin (Invitrogen) coated dishes at day 10 and cultured in DMEM/F12, with 1% (v/v) N2 supplement and 20 ng/mL of FGF-2 (Invitrogen) for additional 4 days.

### Transduction of mouse ES cells by lentivirus

Mouse ES cells were cultured described above. The shRNA lentiviral particles transduction was performed according to the manufacturer's protocol (Santa Cruz). Synemin shRNA lentiviral particles (sc-60526-V, Santa Cruz) and control shRNA lentiviral particles (sc-108080, Santa Cruz) were used for this study. Synemin shRNA (m) Lentiviral Particles (sc-60526, Santa Cruz) is a pool of 3 different shRNA plasmids. Corresponding siRNA sequences are following:

A: Sense GCAAGACUAUGUUUGGAAAtt/Antisense UUUCCAAACAUAGUCUUGCtt

B: Sense GCAAGGCAUUGCCUAUGAAtt/Antisense UUCAUAGGCAAUGCCUUGCtt

C: Sense CAGUCACUCUGGAGUCAAAtt/Antisense UUUGACUCCAGAGUGACUGtt

The transduced mouse ES cell clumps were selected via Puromycin dihydrochloride selection (2 μg/ml), and then analyzed by qRT-PCR, Western blot and immunocytochemistry.

### MTT assay

Different quantities (1 × 10^4^, 2 × 10^4^, 4 × 10^4 ^and 8 × 10^4 ^cells) of three selected synemin knockdown and control clones of mouse ES cells were cultured in 96-well flat-bottomed plates in a triplicate pattern. After eighteen or Forty-two hours, 20 μl 3-(4,5-Dimethylthiazol-2-yl)-2,5-diphenyltetrazolium bromide (MTT) solution (5 mg/ml) was added to each well and incubated for 3.5 h at 37°C. Then media were carefully removed, 200 μl of DMSO was added to each well and the plate was agitated for 10 min at 37°C. Finally, Absorbance of the converted dye of each well is measured at a wavelength of 570 nm.

### RT-PCR analysis

Total RNA from ES cells and mouse embryos was isolated with Trizol Reagent (Invitrogen) and then treated with RNase-free DNase (Qiagen). Aliquots (1 mg) of total RNA were reverse-transcribed into cDNA with first-strand DNA synthesis kit (Roche Diagnostic) following the manufacturer's instructions, and RT-PCR products were amplified as previously described [9]. For quantitative RT-PCR, the PCR analysis was performed with SYBR green PCR technology (Light Cycler 480 system, Roche Diagnostics). Primers were selected with the Primer3 program http://biotools.umassmed.edu. Relative quantification was achieved with the following equation: *R *= 2^ΔCt^target^(control-sample)-ΔCt^ref^(control-sample)^, Ct = cycle threshold http://www.gene-quantification.de. HPRT was used as the reference transcript. Results from 3 independent RT-PCR analyses were expressed as the ratio between mutant and control samples.

Primers for RT-PCR:

Synemin H or M: SynF (5'-AGTCAGGGAGCGTTTCTGTGGACG-3') and SynR (5'-ATCGCTTCTCGTGTCGCTCAAATCC-3');

Synemin L: SynLF (5'-AGAGTGATTGACAGCCTGGAGGAT-3') and SynLR (5'-ACTGTGTGCAATTCTCCAGCCACC-3');

Nestin: NesF (5'-AGGCTTCTCTTGGCTTTCCT-3') and NesR (5'-TGGATCATCAGGGAAGTGGT-3');

GFAP: gfapF (5'-TGGATTTGGAGAGAAAGGTTGAAT-3') and gfapR (5'-CGATAGTGGTTAGCTTCGTGTTTG-3').

Primers for qRT-PCR:

Synemin: F (5'-GGTGGCCTCAGATGAGAAGA-3') and R (5'-GGCTTGCATGTCGGTATTTT-3');

Keratin 8: K8F (5'-TCATCCTATGGGGGACTCAC-3') and K8R (5'-TCTTCACAACCACAGCCTTG-3');

Keratin 18: K18F (5'-CTTGCTGGAGGATGGAGAAG-3') and K18R (5'-GCCTCAGTGCCTCAGAACTC-3')

Oct4: Oct4F (5'-GGGGCTGTATCCTTTCCTCT-3') and Oct4R (5'-GCTGGTGCCTCAGTTTGAAT-3');

Nanog: NanogF (5'-GGACTTTCTGCAGCCTTACG-3') and NanogR (5'-TTTCACCTGGTGGAGTCACA-3');

SOX2: Sox2F (5'-TACCTCTTCCTCCCACTCCA-3') and Sox2R (5'-CTGGGCCATGTGCAGTCTAC-3').

### Production of the antibody against synemin H

A cDNA fragment encoding the exon 4a (formerly named intron IV) of human synemin [4], a specific fragment for synemin H (amino acid residues 1151-1462) was cloned into a pQE32 plasmid (Qiagen). The antibody anti-synemin H was produced as described previously [4]. The resulting rabbit antiserum was characterized by Western blotting analysis. This antibody recognizes both human and mouse synemin H isoforms.

### Immunocytochemistry

ES cells were fixed with cold methanol during 5 minutes, washed with PBS, and then with PBS-Triton X-100 (0.1% v/v). Nonspecific sites were blocked with PBS + 5% (w/v) bovine serum albumin (BSA) during 45 minutes and incubated with primary antibodies for 90 minutes at room temperature (RT). The primary antibodies were polyclonal anti-synemins H/M (1:400), anti-synemin H (1:400) and anti-synemin L (1:100) produced in our laboratory [3], mouse monoclonal antibodies anti-Oct-3/4 (1:100, Santa Cruz), anti-stage-specific embryonic antigen-1 (SSEA-1, 1:100, Chemicon), anti-glial fibrillary acidic protein (GFAP, 1:1000, Sigma), anti-nestin (1:200, Chemicon), anti-β-III tubulin (1:500, Sigma) or rat anti-keratin 8 (TROMA 1, 1:100) [48]. The secondary antibodies used were goat anti-mouse, anti-rat or anti-rabbit immunoglobulins, coupled to the fluorochromes Alex 488 (1:400, Invitrogen) or Cy3 (1:500, Jackson) for 40 minutes at RT. Controls were performed without the primary antibody.

### One- and two-dimensional PAGE and Western blotting

Protein separation by 1D- or 2D-PAGE was carried out as described previously [49,50]. For 2D-PAGE, total protein extract of ES cells was separated by using pH 4-6 gradients IPG strips and 8% SDS/PAGE gels. The proteins were transferred onto nitrocellulose Hybond-C^+ ^or PVDF (GE Healthcare) membranes and nonspecific protein-binding sites were blocked with TBS-T (20 mM Tris-HCl, pH 7.5, 136.8 mM NaCl and 0.1% (v/v) Tween 20) containing 5% (w/v) non-fat milk. The blots were reacted with primary antibodies against synemins H/M (1:3000), synemin L (1:1000), nestin (1:1000), GFAP (1:5000), β-III tubulin (1:3000), Oct-3/4 (1:500), GAPDH (1:5000, Sigma) or α-tubulin (1:5000, Dako) in TBS-T containing 2% (w/v) non-fat milk overnight at 4°C followed by corresponding secondary antibodies anti-rabbit or mouse IgG HRP-conjugated (1:5000; GE Healthcare). The specific binding was revealed by an enhanced chemiluminescence (ECL) detection system (GE Healthcare). For synemin phosphorylation study, the anti-phosphoserine CDKs substrate antibody (1:1000, Cell Signaling) in TBS-T containing 5% (w/v) BSA were used. This antibody detects phosphosérine in a (K/R)(S*)PX(K/R) cyclin-dependent kinase (CDK) motif.

## Authors' contributions

SCSM, ZX and OA carried out the immunocytochemistry and RT-PCR. OA carried out the RT Profiler PCR Array study. SCSM, JCL, ZL and ZX carried out one- and two-dimensional PAGE analysis and Western blotting studies. ZL and ZX carried out anti-synemin H antibody production and analysis. SCSM, ZX, ZL, OA, BP and SR participated the cell culture. ZL, ZX and ND carried out the transduction of synemin, qRT-PCR experiments. ZL and ZX carried out the MTT assay. SCSM, OA, JCL, DP, ZL and ZX analyzed the data. ZX, SCSM, ZL and OA drafted and wrote the manuscript. ZX, ZL, OA and VMN obtained the funding. ZL, ZX, DP and VMN conceived, designed and coordinated the study. All authors read and approved the final manuscript.

## References

[B1] Bellin RM Sernett SW Becker B Ip W Huiatt TW Robson RM Molecular characteristics and interactions of the intermediate filament protein synemin. Interactions with alpha-actinin may anchor synemin-containing heterofilaments J Biol Chem 1999 274 41 29493 29499 10.1074/jbc.274.41.29493 10506213

[B2] Granger BL Lazarides E Synemin: a new high molecular weight protein associated with desmin and vimentin filaments in muscle Cell 1980 22 3 727 738 10.1016/0092-8674(80)90549-8 7006832

[B3] Xue ZG Cheraud Y Brocheriou V Izmiryan A Titeux M Paulin D Li Z The mouse synemin gene encodes three intermediate filament proteins generated by alternative exon usage and different open reading frames Exp Cell Res 2004 298 2 431 444 10.1016/j.yexcr.2004.04.023 15265691

[B4] Titeux M Brocheriou V Xue Z Gao J Pellissier JF Guicheney P Paulin D Li Z Human synemin gene generates splice variants encoding two distinct intermediate filament proteins Eur J Biochem 2001 268 24 6435 6449 10.1046/j.0014-2956.2001.02594.x 11737198

[B5] Sun N Critchley DR Paulin D Li Z Robson RM Identification of a repeated domain within mammalian alpha-synemin that interacts directly with talin Exp Cell Res 2008 314 8 1839 1849 10.1016/j.yexcr.2008.01.034 18342854

[B6] Sun N Critchley DR Paulin D Li Z Robson RM Human alpha-synemin interacts directly with vinculin and metavinculin Biochem J 2008 409 3 657 667 10.1042/BJ20071188 18028034

[B7] Bellin RM Huiatt TW Critchley DR Robson RM Synemin may function to directly link muscle cell intermediate filaments to both myofibrillar Z-lines and costameres J Biol Chem 2001 276 34 32330 32337 10.1074/jbc.M104005200 11418616

[B8] Khanamiryan L Li Z Paulin D Xue Z Self-assembly incompetence of synemin is related to the property of its head and rod domains Biochemistry 2008 47 36 9531 9539 10.1021/bi800912w 18702527

[B9] Izmiryan A Franco CA Paulin D Li Z Xue Z Synemin isoforms during mouse development: multiplicity of partners in vascular and neuronal systems Exp Cell Res 2009 315 5 769 783 10.1016/j.yexcr.2008.12.009 19124017

[B10] Carlsson L Li ZL Paulin D Price MG Breckler J Robson RM Wiche G Thornell LE Differences in the distribution of synemin, paranemin, and plectin in skeletal muscles of wild-type and desmin knock-out mice Histochem Cell Biol 2000 114 1 39 47 10959821 10.1007/s004180000158

[B11] Bhosle RC Michele DE Campbell KP Li Z Robson RM Interactions of intermediate filament protein synemin with dystrophin and utrophin Biochem Biophys Res Commun 2006 346 3 768 777 10.1016/j.bbrc.2006.05.192 16777071

[B12] Hijikata T Nakamura A Isokawa K Imamura M Yuasa K Ishikawa R Kohama K Takeda S Yorifuji H Plectin 1 links intermediate filaments to costameric sarcolemma through beta-synemin, alpha-dystrobrevin and actin J Cell Sci 2008 121 Pt 12 2062 2074 18505798 10.1242/jcs.021634

[B13] Mizuno Y Thompson TG Guyon JR Lidov HG Brosius M Imamura M Ozawa E Watkins SC Kunkel LM Desmuslin, an intermediate filament protein that interacts with alpha-dystrobrevin and desmin Proc Natl Acad Sci USA 2001 98 11 6156 6161 10.1073/pnas.111153298 11353857 PMC33438

[B14] Russell MA Lund LM Haber R McKeegan K Cianciola N Bond M The intermediate filament protein, synemin, is an AKAP in the heart Arch Biochem Biophys 2006 456 2 204 215 10.1016/j.abb.2006.06.010 16934740

[B15] Jing R Pizzolato G Robson RM Gabbiani G Skalli O Intermediate filament protein synemin is present in human reactive and malignant astrocytes and associates with ruffled membranes in astrocytoma cells Glia 2005 50 2 107 120 10.1002/glia.20158 15657940

[B16] Izmiryan A Cheraud Y Khanamiryan L Leterrier JF Federici T Peltekian E Moura-Neto V Paulin D Li Z Xue ZG Different expression of synemin isoforms in glia and neurons during nervous system development Glia 2006 54 3 204 213 10.1002/glia.20378 16817202

[B17] Oshima RG Intermediate filaments: a historical perspective Exp Cell Res 2007 313 10 1981 1994 10.1016/j.yexcr.2007.04.007 17493611 PMC1950476

[B18] Chisholm JC Houliston E Cytokeratin filament assembly in the preimplantation mouse embryo Development 1987 101 3 565 582 2458899 10.1242/dev.101.3.565

[B19] Lu H Hesse M Peters B Magin TM Type II keratins precede type I keratins during early embryonic development Eur J Cell Biol 2005 84 8 709 718 10.1016/j.ejcb.2005.04.001 16180309

[B20] Xue ZG Mounra-Neto V Izmiryan A de Souza Martins SC Larcher JC Paulin D Li Z Ulrich H Intermediate Filament Expression in Mouse Embryonic Stem Cells and Early Embryos Perspectives of Stem Cells: From Tools for Studying Mechanisms of Neuronal Differentiation towards Therapy 2010 Springer Science 59 72

[B21] Brulet P Duprey P Vasseur M Kaghad M Morello D Blanchet P Babinet C Condamine H Jacob F Molecular analysis of the first differentiations in the mouse embryo Cold Spring Harb Symp Quant Biol 1985 50 51 57 2420517 10.1101/sqb.1985.050.01.009

[B22] Jackson BW Grund C Schmid E Burki K Franke WW Illmensee K Formation of cytoskeletal elements during mouse embryogenesis. Intermediate filaments of the cytokeratin type and desmosomes in preimplantation embryos Differentiation 1980 17 3 161 179 10.1111/j.1432-0436.1980.tb01093.x 6161051

[B23] Oshima RG Identification and immunoprecipitation of cytoskeletal proteins from murine extra-embryonic endodermal cells J Biol Chem 1981 256 15 8124 8133 7263644

[B24] Maurer J Nelson B Cecena G Bajpai R Mercola M Terskikh A Oshima RG Contrasting expression of keratins in mouse and human embryonic stem cells PLoS One 2008 3 10 e3451 10.1371/journal.pone.0003451 18941637 PMC2565505

[B25] Constantinescu D Gray HL Sammak PJ Schatten GP Csoka AB Lamin A/C expression is a marker of mouse and human embryonic stem cell differentiation Stem Cells 2006 24 1 177 185 10.1634/stemcells.2004-0159 16179429

[B26] Johnson PJ Tatara A Shiu A Sakiyama-Elbert SE Controlled release of neurotrophin-3 and platelet-derived growth factor from fibrin scaffolds containing neural progenitor cells enhances survival and differentiation into neurons in a subacute model of SCI Cell Transplant 2010 19 1 89 101 10.3727/096368909X477273 19818206 PMC2850943

[B27] Bain G Kitchens D Yao M Huettner JE Gottlieb DI Embryonic stem cells express neuronal properties in vitro Dev Biol 1995 168 2 342 357 10.1006/dbio.1995.1085 7729574

[B28] Guan K Chang H Rolletschek A Wobus AM Embryonic stem cell-derived neurogenesis. Retinoic acid induction and lineage selection of neuronal cells Cell Tissue Res 2001 305 2 171 176 10.1007/s004410100416 11545254

[B29] Lendahl U Zimmerman LB McKay RD CNS stem cells express a new class of intermediate filament protein Cell 1990 60 4 585 595 10.1016/0092-8674(90)90662-X 1689217

[B30] Wiese C Rolletschek A Kania G Blyszczuk P Tarasov KV Tarasova Y Wersto RP Boheler KR Wobus AM Nestin expression--a property of multi-lineage progenitor cells? Cell Mol Life Sci 2004 61 19-20 2510 2522 10.1007/s00018-004-4144-6 15526158 PMC11924557

[B31] Gomes FC Paulin D Moura Neto V Glial fibrillary acidic protein (GFAP): modulation by growth factors and its implication in astrocyte differentiation Braz J Med Biol Res 1999 32 5 619 631 10.1590/S0100-879X1999000500016 10412574

[B32] Garcia-Verdugo JM Doetsch F Wichterle H Lim DA Alvarez-Buylla A Architecture and cell types of the adult subventricular zone: in search of the stem cells J Neurobiol 1998 36 2 234 248 10.1002/(SICI)1097-4695(199808)36:2<234::AID-NEU10>3.0.CO;2-E 9712307

[B33] Alvarez-Buylla A Kohwi M Nguyen TM Merkle FT The heterogeneity of adult neural stem cells and the emerging complexity of their niche Cold Spring Harb Symp Quant Biol 2008 73 357 365 10.1101/sqb.2008.73.019 19022766

[B34] Bramanti V Tomassoni D Avitabile M Amenta F Avola R Biomarkers of glial cell proliferation and differentiation in culture Front Biosci (Schol Ed) 2010 2 558 570 10.2741/s85 20036968

[B35] Doetsch F A niche for adult neural stem cells Curr Opin Genet Dev 2003 13 5 543 550 10.1016/j.gde.2003.08.012 14550422

[B36] Chambers I Colby D Robertson M Nichols J Lee S Tweedie S Smith A Functional expression cloning of Nanog, a pluripotency sustaining factor in embryonic stem cells Cell 2003 113 5 643 655 10.1016/S0092-8674(03)00392-1 12787505

[B37] Mitsui K Tokuzawa Y Itoh H Segawa K Murakami M Takahashi K Maruyama M Maeda M Yamanaka S The homeoprotein Nanog is required for maintenance of pluripotency in mouse epiblast and ES cells Cell 2003 113 5 631 642 10.1016/S0092-8674(03)00393-3 12787504

[B38] Avilion AA Nicolis SK Pevny LH Perez L Vivian N Lovell-Badge R Multipotent cell lineages in early mouse development depend on SOX2 function Genes Dev 2003 17 1 126 140 10.1101/gad.224503 12514105 PMC195970

[B39] Friel R van der Sar S Mee PJ Embryonic stem cells: understanding their history, cell biology and signalling Adv Drug Deliv Rev 2005 57 13 1894 1903 10.1016/j.addr.2005.08.002 16271417

[B40] Loh YH Wu Q Chew JL Vega VB Zhang W Chen X Bourque G George J Leong B Liu J The Oct4 and Nanog transcription network regulates pluripotency in mouse embryonic stem cells Nat Genet 2006 38 4 431 440 10.1038/ng1760 16518401

[B41] Rodda DJ Chew JL Lim LH Loh YH Wang B Ng HH Robson P Transcriptional regulation of nanog by OCT4 and SOX2 J Biol Chem 2005 280 26 24731 24737 10.1074/jbc.M502573200 15860457

[B42] Furusawa T Ikeda M Inoue F Ohkoshi K Hamano T Tokunaga T Gene expression profiling of mouse embryonic stem cell subpopulations Biol Reprod 2006 75 4 555 561 10.1095/biolreprod.105.049502 16687650

[B43] Maro B Kubiak J Gueth C De Pennart H Houliston E Weber M Antony C Aghion J Cytoskeleton organization during oogenesis, fertilization and preimplantation development of the mouse Int J Dev Biol 1990 34 1 127 137 2203452

[B44] Zehner ZE Regulation of intermediate filament gene expression Curr Opin Cell Biol 1991 3 1 67 74 10.1016/0955-0674(91)90167-W 1854486

[B45] Omary MB Ku NO Tao GZ Toivola DM Liao J "Heads and tails" of intermediate filament phosphorylation: multiple sites and functional insights Trends Biochem Sci 2006 31 7 383 394 10.1016/j.tibs.2006.05.008 16782342

[B46] Sandoval IV Colaco CA Lazarides E Purification of the intermediate filament-associated protein, synemin, from chicken smooth muscle. Studies on its physicochemical properties, interaction with desmin, and phosphorylation J Biol Chem 1983 258 4 2568 2576 6822575

[B47] Faherty S Fitzgerald A Keohan M Quinlan LR Self-renewal and differentiation of mouse embryonic stem cells as measured by Oct4 expression: the role of the cAMP/PKA pathway In Vitro Cell Dev Biol Anim 2007 43 1 37 47 10.1007/s11626-006-9001-5 17570033

[B48] Sukoyan MA Kerkis AY Mello MR Kerkis IE Visintin JA Pereira LV Establishment of new murine embryonic stem cell lines for the generation of mouse models of human genetic diseases Braz J Med Biol Res 2002 35 5 535 542 12011937 10.1590/s0100-879x2002000500004

[B49] Bonnet C Boucher D Lazereg S Pedrotti B Islam K Denoulet P Larcher JC Differential binding regulation of microtubule-associated proteins MAP1A, MAP1B, and MAP2 by tubulin polyglutamylation J Biol Chem 2001 276 16 12839 12848 10.1074/jbc.M011380200 11278895

[B50] de Souza Martins SC Romao LF Faria JC de Holanda Afonso RC Murray SA Pellizzon CH Mercer JA Cameron LC Moura-Neto V Effect of thyroid hormone T3 on myosin-Va expression in the central nervous system Brain Res 2009 1275 1 9 19379719 10.1016/j.brainres.2009.03.070

